# The *Mycobacterium tuberculosis* ClpP1P2 Protease Interacts Asymmetrically with Its ATPase Partners ClpX and ClpC1

**DOI:** 10.1371/journal.pone.0125345

**Published:** 2015-05-01

**Authors:** Julia Leodolter, Jannis Warweg, Eilika Weber-Ban

**Affiliations:** Institute for Molecular Biology & Biophysics, ETH Zurich, Zurich, Switzerland; University of the Basque Country, SPAIN

## Abstract

Clp chaperone-proteases are cylindrical complexes built from ATP-dependent chaperone rings that stack onto a proteolytic ClpP double-ring core to carry out substrate protein degradation. Interaction of the ClpP particle with the chaperone is mediated by an N-terminal loop and a hydrophobic surface patch on the ClpP ring surface. In contrast to *E*. *coli*, *Mycobacterium tuberculosis* harbors not only one but two ClpP protease subunits, ClpP1 and ClpP2, and a homo-heptameric ring of each assembles to form the ClpP1P2 double-ring core. Consequently, this hetero double-ring presents two different potential binding surfaces for the interaction with the chaperones ClpX and ClpC1. To investigate whether ClpX or ClpC1 might preferentially interact with one or the other double-ring face, we mutated the hydrophobic chaperone-interaction patch on either ClpP1 or ClpP2, generating ClpP1P2 particles that are defective in one of the two binding patches and thereby in their ability to interact with their chaperone partners. Using chaperone-mediated degradation of ssrA-tagged model substrates, we show that both *Mycobacterium tuberculosis* Clp chaperones require the intact interaction face of ClpP2 to support degradation, resulting in an asymmetric complex where chaperones only bind to the ClpP2 side of the proteolytic core. This sets the Clp proteases of *Mycobacterium tuberculosis*, and probably other Actinobacteria, apart from the well-studied *E*. *coli* system, where chaperones bind to both sides of the protease core, and it frees the ClpP1 interaction interface for putative new binding partners.

## Introduction

*Mycobacterium tuberculosis* (Mtb) is a gram-positive bacterium of the phylum Actinobacteria and the causative agent of tuberculosis. More and more strains are evolving resistance to the antibiotics currently in use [1, 2], but several new and promising compounds have been discovered in recent years (acyldepsipeptides [3–5], β-lactones [6], cyclomarin A [7], lassomycin [8]), all targeting the Clp (caseinolytic protease) chaperone-protease system. The Clp chaperone-protease is a bacterial multi-subunit protein complex involved in intracellular protein degradation. It is active in general protein quality control as well as specific degradation of proteins participating in regulatory processes [9–11]. The best described substrate class comprises proteins tagged with the ssrA tag, a short peptide sequence C-terminally added to proteins by the tmRNA system to rescue stalled ribosomes [12, 13]. To mediate substrate degradation, the Clp chaperone-proteases form cylindrical complexes built from rings of protease and chaperone subunits stacked on top of one another. The core of the structure consists of the ClpP proteolytic subunits that assemble into a double-ring stack of two heptameric rings enclosing a sequestered space [14]. The protease active sites line the inside of this chamber and are made up of the Ser-His-Asp catalytic triad typical for serine proteases. The access to the chamber is controlled by hexameric unfoldases (ClpX, ClpA or ClpC) of the AAA+ type (**A**TPase **a**ssociated with various cellular **a**ctivities) that recognize substrates, unfold them in an ATP-dependent manner and thread them into the proteolytic chamber [15, 16]. Two conserved interaction elements on the protease particle are involved in the association with the chaperones. One interaction feature is an N-terminal loop positioned at the axial pore (N-loop), where substrates pass from the chaperone to the protease [17–19]. The other interaction feature is a hydrophobic patch located on the face of the protease ring to which binds a loop of the chaperone containing a conserved LGF-motif in case of the Mtb chaperones ClpX and ClpC1 (LGF-loop) [14, 20, 21]. Interestingly, antibiotics of the acyldepsipeptide (ADEP) class bind to this patch in place of the LGF-loop and deregulate the Clp protease by mimicking chaperone binding [22–24].

In contrast to the well-studied Clp system of *E*. *coli*, Actinobacteria contain not only one ClpP subunit, but carry two or more homologous genes. The Mtb system harbors two ClpP genes, *clpP1* and *clpP2*, which are co-expressed from one operon [25]. Both subunits are essential [26, 27] and both are necessary for the activity of the complex [28]. Like other ClpP protease subunits, Mtb ClpP1 and ClpP2 carry N-terminal propeptides that are cleaved off in a processing step, as has been demonstrated *in vivo* [29, 30]. To produce the mature, functional ClpP particle, ClpP1 and ClpP2 assemble from one homoheptameric ring of each subunit into a ClpP1P2 hetero double-ring. So far, this assembly has only been observed *in vitro* in the presence of a synthetic non-natural activator peptide [24, 30]. This activator peptide is an N-blocked dipeptide, usually Z-Leu-Leu (Benzyloxycarbonyl-L-Leucyl-L-Leucine), Z-Leu-Leu-H (Benzyloxycarbonyl-L-Leucyl-L-Leucinal) or a similar molecule, that binds near the active sites of the proteolytic particle and stabilizes the active conformation of the ClpP1P2 double-ring [24, 30]. This functional conformation of the complex is also stabilized by the presence of the Mtb chaperone ClpX and protein substrate, acting synergistically with the activator peptide [31]. ClpC1 also appears to have a stabilizing effect, although here ClpC1 of *M*. *smegmatis* was used in combination with Mtb ClpP1P2 [31]. Interestingly, *E*. *coli* ClpX rings (EcClpX) can interact with the Mtb ClpP1P2 complex and even promote substrate degradation more than ten-fold faster compared to the Mtb chaperones [31].

In the *E*. *coli* ClpAP and ClpXP complexes, chaperone binding to both sides of the ClpP cylinder was shown to be the preferred state [32, 33]. However, the *E*. *coli* ClpP double-ring is a symmetric particle made of 14 identical subunits resulting in two equal binding surfaces, a situation fundamentally different from that in the Mtb ClpP1P2 double-ring, which presents a different binding surface on each face of the cylinder. The Mtb ClpP1P2 double-ring structure shows considerable differences between its two potential chaperone-interaction faces [24] which could indicate that specific chaperone binding to one face or the other occurs.

Here, we investigate the effects of the asymmetry of the Mtb ClpP1P2 hetero double-ring particle on its assembly, propeptide processing and interaction with the ATP-dependent unfoldases ClpX and ClpC1 of Mtb. We show that assembly of ClpP1P2 is independent of the presence of the propeptide and we provide *in vitro* evidence that an asymmetric behavior is already apparent during the propeptide processing reaction, with ClpP1 as the main carrier of the processing activity. To investigate whether ClpP1 and ClpP2 exhibit differences in interaction with the chaperones ClpX and ClpC1, we designed variants of both proteins impaired in chaperone interaction by mutating the hydrophobic patch necessary for LGF-loop binding. Our results show that both chaperones need the intact interaction face of ClpP2 to support chaperone-mediated substrate degradation, suggesting that the ClpP2 ring face is the sole interaction platform for these chaperones.

## Materials and Methods

### Alignment

Protein sequences of *M*. *tuberculosis* (Mtb) ClpP1 (P9WPC5), Mtb ClpP2 (P9WPC3) and their actinobacterial homologues, as well as *E*. *coli* ClpP (P0A6G7), were extracted from the Uniprot database (http://www.uniprot.org/) and aligned using the Clustal Omega algorithm [34, 35]. Visualisation was performed using Jalview [36].

### Cloning, expression and protein purification

All genes were amplified by PCR with Phusion DNA polymerase (New England Biolabs) from *M*. *tuberculosis* H37Rv genomic DNA. N-terminal deletions and active-site mutations of *clpP1* and *clpP2* were introduced by site-directed mutagenesis.*ClpP1* and *clpP2*, containing a C-terminal His_4_-tag, were separately ligated into the pET-Duet-1 coexpression plasmid. *ClpX* was fused to a C-terminal Tobacco Etch Virus (TEV) endopeptidase cleavage site followed by GFP-His_6_ in a pET24 vector. *ClpC1* was ligated into a p7XC3H FX vector [37], including a stop codon to produce the untagged protein. The malate dehydrogenase (*mdh*) gene with the Mtb ssrA-tag (AADSHQRDYALAA) added C-terminally was ligated into a pET20 vector. GFP-ssrA was recloned from the construct used in [38], with the *E*. *coli* ssrA-tag changed to the Mtb ssrA-tag sequence. Correct insertion and sequences of the genes were verified by sequencing.

All constructs were transformed into *E*. *coli* Rosetta (DE3) (Invitrogen) and grown in LB broth supplemented with the respective antibiotic. Expression was induced at an OD_600_ of 0.8 by addition of 0.1 mM IPTG and expression was carried out overnight at 20°C. Cells were cracked by sonication or with a microfluidizer. The ClpP1 and ClpP2 proteins and variants were purified by Ni-NTA affinity chromatography and subsequent gel filtration on a Superose 6 column (GE Healthcare) in Buffer A (50 mM Hepes-NaOH pH 7.5, 300 mM NaCl, 10% glycerol). ClpX and MDH-ssrA were purified by Ni-NTA affinity chromatography, TEV cleavage and a reverse Ni-NTA step, followed by dialysis into Buffer A. ClpC1 was purified on an anion exchange Fast Flow Q column, followed by ammonium sulfate (AS) precipitation and a Superdex 200 (GE Healthcare) gel filtration step. The final fractions were again precipitated by AS, then resuspended and dialysed into Buffer A. GFP-ssrA, with either the Mtb or *E*. *coli* ssrA-tag was purified as described [38]. EcClpX was expressed and purified as described [32]. ClpX and ClpC1 were further dialysed into Buffer J (50 mM Hepes-KOH pH 7.5, 150 mM KCl, 15% glycerol, 20 mM MgCl_2_). Correct molecular mass of the final proteins was verified by mass spectrometry.

### Analytical gel filtration

Analytical gel filtrations at room temperature were performed on a Superdex 200 10/300 GL column (GE Healthcare, 24 ml) in Buffer A at a flow rate of 1 ml/min on an ÄKTA Purifier System. Prior to loading, the sample (25 μM ClpP1 and/or ClpP2 protomer) was centrifuged in an Eppendorf table-top centrifuge for 10 minutes at 13’000 rpm. 100 μl of the sample were injected onto the column. Proteins were detected by absorption at 280 nm. For analytical gel filtration runs at 4°C the same Superdex 200 column was used in Buffer A with a flowrate of 0.6 ml/min on a different ÄKTA Purifier. The column was calibrated at room temperature and at 4°C with the same set of standard proteins (GE Healthcare Gel filtration Calibration Kit), and a calibration curve was calculated for each.

### Processing of the N-terminal ClpP propeptides

For processing assays of the N-terminal propeptides in the presence of the activator Z-Leu-Leu-H (Benzyloxycarbonyl-L-Leucyl-L-Leucinal) (PeptaNova), 25 μM full-length ClpP1 and ClpP2 protomer each (referred to as proClpP1 and proClpP2) were first incubated for 1 hour at room temperature in Buffer A without the activator. Then the processing reaction was started by the addition of 1 mM activator dissolved in DMSO, resulting in a final DMSO concentration of 2%. Alternatively, for processing in presence of 1 μM ClpC1 hexamer and 10 μM GFP-ssrA or 1 μM ClpX hexamer and 10 μM MDH-ssrA, the reaction was performed in Buffer J, pH 7 or 7.5, respectively, supplemented with 5 mM ATP, 1 mM DTT, 40 mM phosphocreatine and 1 U/ml creatine phosphokinase, with 0.5 μM preassembled proClpP1P2 double-ring. The reaction was stopped at the indicated time points by the addition of Laemmli buffer and the samples were heated for 10 minutes at 95°C. The samples were analyzed on a 15% SDS-PA gel. For the production of mature ClpP1P2 for use in further biochemical assays, proClpP1 and proClpP2 were incubated at a concentration of 50 μM (for wild-type) or 70 μM (for hydrophobic patch variants) protomer each in Buffer A overnight at room temperature in the presence of 1 mM activator. Processing and assembly were verified by SDS-PAGE and analytical gel filtration. The mature ClpP1P2 complex was separated from the activator using a PD-10 desalting column (GE Healthcare).

### Degradation of MDH-ssrA by ClpXP1P2

Protein degradation assays using MDH-ssrA as a model substrate were performed at room temperature in Buffer J with 1 μM ClpX hexamer, 0.5 μM mature ClpP1P2 double-ring complex preassembled for 1 hour at room temperature, 2 μM MDH-ssrA, 5 mM ATP, 1 mM DTT, 20 mM phosphocreatine and 1 U/ml creatine phosphokinase. The reaction was stopped at the indicated time points by the addition of Laemmli buffer and the samples were heated for 10 minutes at 95°C. The samples were analyzed on a 15% SDS-PA gel.

### Degradation of GFP-ssrA by ClpC1P1P2 and EcClpXP1P2

Protein degradation assays using GFP-ssrA as a model substrate were carried out by following the loss of the GFP fluorescence signal. The experiments were performed in a BioTek Synergy 2 plate reader with a Tungsten light source, with an excitation wavelength of 360/40 nm and emission wavelength of 528/20 nm (50% optics position, sensitivity: 90) in Corning non-binding 96-well half area assay plates in 50 μl reaction volume. Reaction conditions were the same as given for the degradation of MDH-ssrA, but using ClpC1 and GFP-ssrA (Mtb ssrA sequence) instead of ClpX and MDH-ssrA, respectively. For the reaction with ClpC1, the pH of the reaction buffer was adjusted to 7. Before and after the reaction, samples were drawn for SDS-PAGE analysis. Reaction buffer conditions for the degradation of GFP-ssrA *(E*. *coli* ssrA sequence) by EcClpX and ClpP1P2 were the same as for Mtb ClpXP1P2.

### Negative stain Electron Microscopy

1.4 μM proClpP1 protomer (200 nM heptamer) sample was applied to a carbon-coated copper mesh grid for 20 seconds and subsequently stained with 2% aqueous uranyl acetate for 2 minutes. Imaging was perfomed in a FEI Morgagni 268 transmission electron microscope operating at 100 kV.

## Results

### The ClpP1P2 double-ring complex can be assembled without activator peptide and is processed in a chaperone-dependent manner

**Fig 1:**
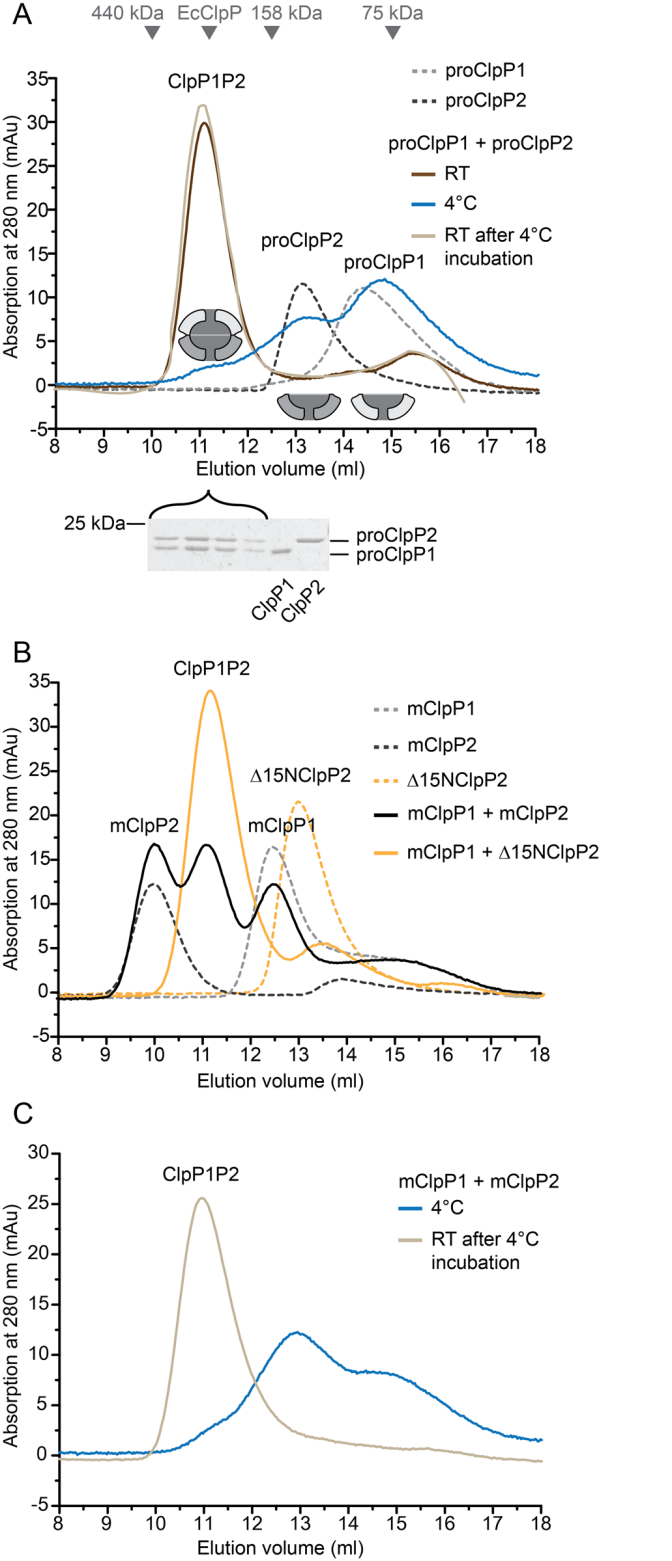
Assembly of ClpP1 and ClpP2 to the ClpP1P2 double-ring particle. Analytical gel filtration runs were performed on a Superdex 200 gel filtration column (24 ml). Unless noted otherwise, all runs were performed at room temperature. The assembly state of the proteins is indicated by a cartoon depiction in the topmost graph of either single rings in light grey for ClpP1 and dark grey for ClpP2, or the assembled double-ring particle. Molecular size markers are indicated as arrow tips above the elution profiles in the first graph. A marker for *E*. *coli* ClpP (EcClpP) is included as a reference for the double-ring assembly. The concentration of ClpP1 and ClpP2 is always 25 μM protomer. A. ClpP1 and ClpP2 containing the N-terminal propeptide (proClpP1, proClpP2) assemble to double-ring complexes in the absence of the activator. When proClpP1 (dashed light grey) and proClpP2 (dashed dark grey) are incubated together at room temperature overnight they assemble into the double-ring complex (dark brown). SDS-PAGE shows a 1:1 ratio of ClpP1 to ClpP2 in the peak fraction. Incubation of the assembled complex at 4°C for 3 hours leads to disassembly (blue). Reincubation of the complex at room temperature for 3 hours leads to reassembly into the double-ring complex (light brown). B. Analytical gel filtration of individually loaded mature ClpP1 (mClpP1, dashed light grey), mature ClpP2 (mClpP2, dashed dark grey) and Δ15NClpP2 (dashed orange). mClpP2 shows its main elution peak at 10 ml corresponding to a size of around 450 kDa. Elution profiles were also recorded after overnight incubation of mClpP1 and mClpP2 (black), and of mClpP1 and Δ15NClpP2 (orange). C. Elution profiles of *in vitro* processed mClpP1P2. To produce the mature complex proClpP1 and proClpP2 were incubated in the presence of 1 mM activator overnight at room temperature. The activator was then removed by buffer exchange and the mClpP1P2 complex was incubated at 4°C for 3 hours leading to disassembly of the complex (blue). Reincubation of the complex at room temperature for 3 hours leads to reassembly into the double-ring complex (light brown).

The mycobacterial ClpP protease subunits ClpP1 and ClpP2 were previously shown to each form a homo-heptameric ring that assembles with the other into the proteolytically active ClpP1P2 double-ring complex in the presence of synthetic activator peptides [24, 30]. To investigate the assembly behavior of ClpP1 and ClpP2 into the double-ring particle in absence of any activator peptide, we carried out analytical gel filtration analysis, both at room temperature and at 4°C. Protease subunits with the propeptides still present were used (proClpP1 and proClpP2), which corresponds most closely to the situation encountered after the subunits are first translated in the cell and are coming together in the initial assembly of the ClpP1P2 complex. At room temperature, recombinant proClpP1 and proClpP2 assemble into the double ring complex in absence of the activator, as shown in analytical gel filtration by an elution shift of the peaks of the single proClpP1 and proClpP2 rings (Fig 1A, dashed light and dark grey traces) to the peak of the double-ring complex at ~11 ml (Fig 1A, dark brown trace). Based on a calibration curve using molecular weight standards this elution volume corresponds to ~300 kDa, which is equivalent to the size of the assembled double-ring particle. The *E*. *coli* ClpP double-ring elutes at this position (EcClpP, indicated by a grey arrow tip), further supporting a double-ring assembly state. In addition, SDS-PAGE analysis confirms that fractions collected from this elution peak contain equimolar amounts of ClpP1 and ClpP2 (Fig 1A, gel slice). When the two ClpP subunits are run separately, they elute at positions corresponding to lower molecular weight than the double-ring complex. However, while ClpP2 elutes roughly at the expected position of a single ring, ClpP1 elutes even later despite the similar subunit size. To ensure that ClpP1 nevertheless forms rings on its own, we analyzed the ClpP1 sample by negative stain electron microscopy. Top views of ring-shaped complexes with a stain-filled center are clearly visible, indicating that ClpP1 is assembled (S1 Fig). The lower elution volume could be due to unspecific interactions with the column material, potentially involving the propeptide, since the mature ClpP1 (mClpP1) runs at the expected elution volume close to ClpP2 (Fig 1B). It could also indicate a tendency of a portion of the single rings to dissociate into smaller oligomeric states [29].

After incubation of the assembled complex at 4°C, ClpP1P2 double-rings disassemble into single rings as shown by subsequent gel filtration at 4°C (Fig 1A, blue trace). The disassembly is reversible, since reincubation at room temperature and subsequent analysis by gel filtration shows a single main elution peak at the double-ring particle position (Fig 1A, light brown trace). This shows that the assembly of the ClpP1 and ClpP2 single rings to the ClpP1P2 double-ring particle occurs also in absence of the activator. It is, however, temperature-dependent and does not occur quantitatively at low temperature.

In the functional ClpP1P2 complex the propeptides are processed to form the mature particle [29, 30]. Previous studies had identified the processing site to be located between Ala12 and Arg13 for the Mtb ClpP2 subunit [29, 30]. For the propeptide cleavage in ClpP1, two different cleavage sites were reported by two different studies both expressing ClpP1 in absence of ClpP2, namely between Arg8 and Ser9 [29] or between Asp6 and Met7 [30]. Using *in vitro* processing of the propeptide-containing ClpP1P2 double-ring complex in presence of activator peptide, we predominantly obtained cleavage between Met7 and Arg8 (S2 Fig). Therefore, to test whether the mature particle can be generated *in vitro* by mixing recombinantly produced ClpP subunits lacking the propeptide and to test if the propeptides might be important for assembly, we produced mature ClpP1 and ClpP2 (mClpP1, mClpP2), where the residues corresponding to the propeptides (the first 6 and 12 residues, respectively) were removed. Methionine 7 was kept in the mature ClpP1 construct, because it forms the translation start site for expression. Interestingly, while mClpP1 eluted at a position corresponding to a size of ~150 kDa and thus one heptameric ring (Fig 1B, dashed light grey trace), mClpP2 eluted much earlier, in fact even earlier than the double-ring particle (Fig 1B, dashed dark grey trace), translating into an apparent molecular mass of ~450 kDa. This could correspond to a non-native assembly state of three stacked heptameric rings, a behavior observed previously by Benaroudj *et al* [29]. Likely due to this non-native stacking, only a fraction of mixed mClpP1 and mClpP2 assembles into ClpP1P2 double-ring particles (Fig 1B, black trace).

To circumvent the aggregation tendencies of mClpP2 and still be able to test the role of the propeptides in assembly, we generated a variant of mClpP2 that was shortened by another three residues beyond the processing site (Δ15NClpP2). This variant is soluble and runs at the position expected for the ClpP2 single ring (Fig 1B, dashed orange trace). Upon incubation with mClpP1, the double-ring particle is formed with the expected elution properties (Fig 1B, orange trace), indicating that the propeptides do not play an active role in ClpP1P2 particle assembly.

The temperature dependence of the mature double-ring complex resembles that of the propeptide-containing complex. When mature ClpP1P2 double-ring particle is generated by addition of activator peptide (see next paragraph for processing) and is then incubated at 4°C, it also disassembles into single rings and reassembles at room temperature (Fig 1C, blue and light brown trace).

**Fig 2:**
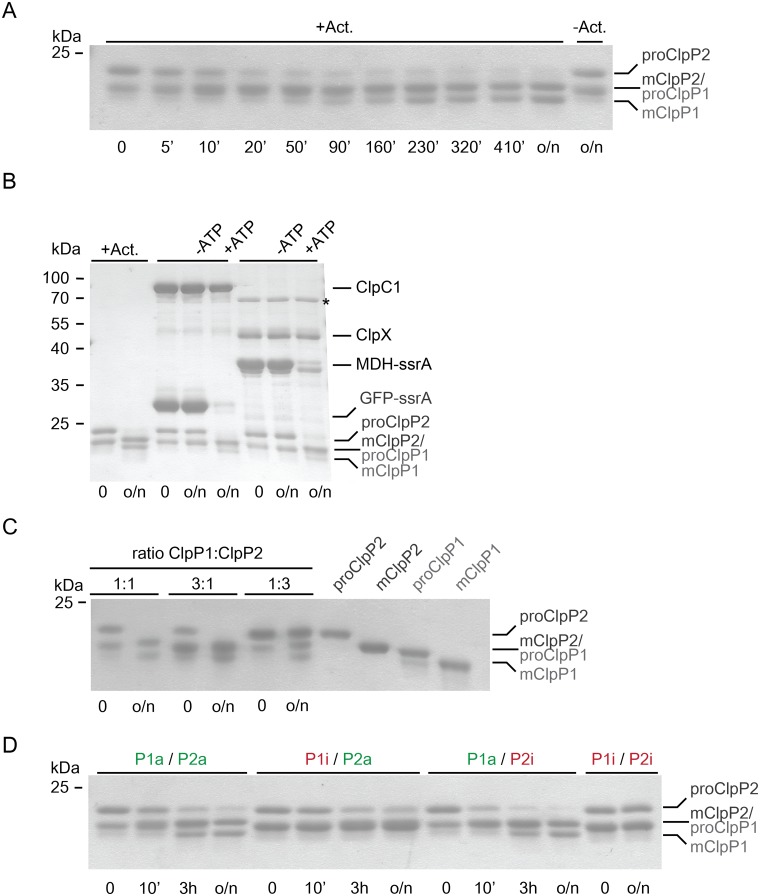
Propeptide processing of ClpP1 and ClpP2 in the ClpP1P2 double-ring assembly. Maturation of double-ring assembled ClpP1 and ClpP2 carrying the N-terminal propeptide (proClpP1, proClp2) to the mature proteins (mClpP1, mClpP2) was performed at room temperature and followed by SDS-PAGE analysis. Samples were taken at the indicated time points. A. Propeptide processing of ClpP1 and ClpP2 (25 μM protomer each), in the presence of 1 mM activator (+Act.), or without activator (−Act.). B. Propeptide processing of ClpP1 and ClpP2 (0.5 μM double-ring particle) in the absence of activator and the presence of 1 μM ClpC1 hexamer, 10 μM GFP-ssrA or 1 μM ClpX hexamer and 10 μM MDH-ssrA, and 5mM ATP (+ATP) or without ATP (−ATP). For comparison, overnight processing of ClpP1 and ClpP2 in the presence of 1 mM activator is shown. The asterisk (*) indicates a protein contaminant that was identified by mass spectrometry to contain DnaK from *E*. *coli*. C. Propeptide processing of ClpP1 and ClpP2 at different molar ratios of ClpP1:ClpP2 with 25 and 75 μM, respectively, ClpP1 or ClpP2 protomer concentration in the presence of 1 mM activator. D. Propeptide processing of double-ring complexes assembled from wild-type (active) ClpP1 and ClpP2 (P1a, P2a, 25 μM protomer each) and protease-inactive ClpP1^S98A^, ClpP2^S110A^ (P1i, P2i) in the presence of 1 mM activator.

To generate the mature ClpP1P2 complex from proClpP1 and proClpP2, the propeptides at their N-termini have to be cleaved off. Formation of the double-ring is a requirement for propeptide processing to occur, since the individual subunits alone, even in presence of activator peptide, show no peptidase activity [30]. Processing of the propeptides is the first activity performed by the newly formed ClpP1P2 complex. To investigate this activity and to assess a potential asymmetry across the ClpP1:ClpP2 ring-ring interface in the propeptide processing reaction, we analyzed the propeptide cleavage activity in the assembled double-ring particle. To follow the processing of individual subunits in the wild-type particle, we first incubated proClpP1 and proClpP2 together for one hour at room temperature resulting in the assembly of the unprocessed ClpP1P2 complex. Processing was allowed to occur during overnight incubation either in presence or absence of the activator peptide Z-Leu-Leu-H, from here on referred to as activator. For the reaction in absence of the activator, only an end-point sample was drawn after overnight incubation (Fig 2A, last lane). For the sample in presence of the activator, the progress of the cleavage reaction was followed at specific time intervals by drawing samples that were then analyzed by SDS-PAGE (Fig 2A). Unfortunately, the generated mClpP2 overlays with the band of proClpP1 and is then not visible as a separate band. Therefore, processing of ClpP2 is followed by assessing the disappearance of the proClpP2 band and processing of ClpP1 is followed by assessing the appearance of the mClpP1 band. After the first 20 minutes of processing, the band for proClpP2 is considerably reduced while on the other hand hardly any mature ClpP1 has yet been produced. This suggests that the ClpP2 propeptide is processed first. While we showed that assembly to the double-ring particle occurs in absence of the activator, this experiment demonstrates that maturation to the processed complex requires the presence of the activator (Fig 2A). As the synthetic activator is not present *in vivo*, this raises the question which endogenous molecules could serve this function. Inside the cell, ClpP particles form assemblies with their chaperone partners. To test whether processing could occur in the absence of the activator as long as a chaperone partner and a protein substrate are present, we incubated proClpP1 and proClpP2 in the presence of the chaperone ClpC1 and the model substrate GFP-ssrA, or the chaperone ClpX and the model substrate MDH-ssrA. In Fig 2B we show that, indeed, processing of proClpP1 and proClpP2 to mClpP1 and mClpP2 occurs when the chaperone ClpX or ClpC1 and a degradation substrate is present. The processing in this case is dependent on ATP hydrolysis, suggesting that substrate must enter the proteolytic particle. Therefore, *in vivo*, the natural interaction partners of the ClpP particle likely serve to activate propeptide processing. As processing in presence of the activator provided a cleaner experimental setup (Fig 2B, +Act), we made use of the activator as a tool for further *in vitro* processing assays.

Given that propeptide cleavage occurs only in the assembled double-ring particle, processing of an excess of one subunit over the other would require dissociation followed by reassociation with the unprocessed subunits. Therefore, the degree of processing of excess ClpP subunits can be used as a measure for the kinetic stability of the complex. When the two subunits are provided in equimolar amounts, overnight incubation with activator results in complete processing of both subunits (Fig 2C, first two lanes). Providing proClpP1 in three-fold excess under the same conditions results in processing of only a fraction of proClpP1 to mClpP1 (Fig 2C, lanes 3 and 4). The unfortunate overlap between the bands of mClpP2 and proClpP1 makes it difficult to assess the exact amount of proClpP1 left after overnight incubation. However, it is clear that the mClpP1 band would have to be three times more intense than the mClpP2/proClpP1 band, if complete processing occurred. This is not the case. Likewise, with proClpP2 in three-fold excess, surplus proClpP2 is not processed and only equimolar bands for mClpP1 and mClpP2 are observed (Fig 2C, lanes 5 and 6). These results demonstrate that the processed ClpP1P2 double-ring particle is kinetically very stable.

### ClpP1 is the main actor in ClpP1P2 double-ring processing

For the ClpP particle of *Streptomyces lividans*, an organism that is related to Mtb and also encodes ClpP1 and ClpP2 subunits, it was suggested based on *in vivo* experiments that processing of the propeptides occurs across the ClpP1P2 double-ring interface [39]. For Mtb ClpP1 and ClpP2, processing was observed for either subunit expressed on its own in *M*. *smegmatis* [30]. When both proteins were separately expressed in *E*. *coli* and purified, only active ClpP1 showed a double band indicative of processing, while ClpP2 only showed a double band when active ClpP1 but not when inactive ClpP1 was coexpressed [29]. The reports are partly contradictory and in both cases processing activity was assayed during expression in heterologous hosts where interference from endogenous proteins could occur. Furthermore, the latter study stated that ClpP1P2 tetradecamers containing both ClpP1 and ClpP2 are not formed and only partial processing was observed [29]. Therefore, to investigate this in the background of the functionally relevant hetero oligomer and to exclude possible host interference, we produced mixed active and inactive ClpP1P2 complexes that were then tested for propeptide processing in an *in vitro* setup. Four different double-ring particles were tested, namely particles with both ClpP rings active (P1a/P2a) or both inactive (P1i/P2i) with the active-site serine mutated to alanine (ClpP1^S98A^, ClpP2^S110A^), or mixed particles, where either the ClpP1 or the ClpP2 subunit was in the inactive (i) and the other in the active (a) form (P1i/P2a, P1a/P2i). Processing was initiated by adding activator and the reaction was followed by SDS-PAGE analysis (Fig 2D). As expected, when both ClpP1 and ClpP2 subunits are active (P1a/P2a), the fully mature particle with mClpP1 and mClpP2 is produced, while inactivation of both ClpP1 and ClpP2 (P1i/P2i) abolishes processing entirely. When a catalytically active ClpP1 ring was combined with a catalytically inactive ClpP2 ring (P1a/P2i), processing of both rings was complete and even occurred somewhat faster than with wt complex. However, when only ClpP2 was active (P1i/P2a), processing was significantly impaired. Only a fraction of ClpP2 was processed after overnight incubation and no ClpP1 processing was observed. This indicates that while ClpP1 in context of the ClpP1P2 particle can process both itself and ClpP2, ClpP2 is unable to process the trans-ring. Furthermore ClpP2 exhibits little activity even towards its own propeptide, suggesting that ClpP1 is the main propeptide processor of the ClpP1P2 double-ring particle.

### ClpP2 is the main interaction platform for the ATPase rings

ClpP1 and ClpP2 show specialization in terms of their processing activity (i.e. ClpP1 is almost solely responsible for processing), and they were previously shown to exhibit different substrate cleavage specificities [30, 40]. However, in the case of substrate degradation, this specialization is not apparent, as protease inactivation of either subunit did not significantly slow down *in vitro* protein degradation (in case of ClpX-dependent degradation), or even enhanced its rate (in case of the heterologous ^Msm^ClpC1^Mtb^ClpP1P2 complex) [31]. It is unlikely that the only asymmetric behavior in ClpP1 and ClpP2 would be in their processing activity, as differences between the two molecules are not only apparent in the active-site substrate binding cleft, but also in the N-terminal loop region as well as the hydrophobic patch, both of which were shown in other bacterial ClpP particles to be involved in chaperone interaction [14, 17, 24]. We were interested to find out whether the asymmetry in the ClpP1P2 protease particle also translates into an asymmetry of chaperone binding. Do both chaperones, ClpX and ClpC1, bind to ClpP1 and ClpP2, or is the interaction of one chaperone restricted to one protease partner, e.g. binding of ClpX to ClpP2 or to ClpP1 only?

**Fig 3:**
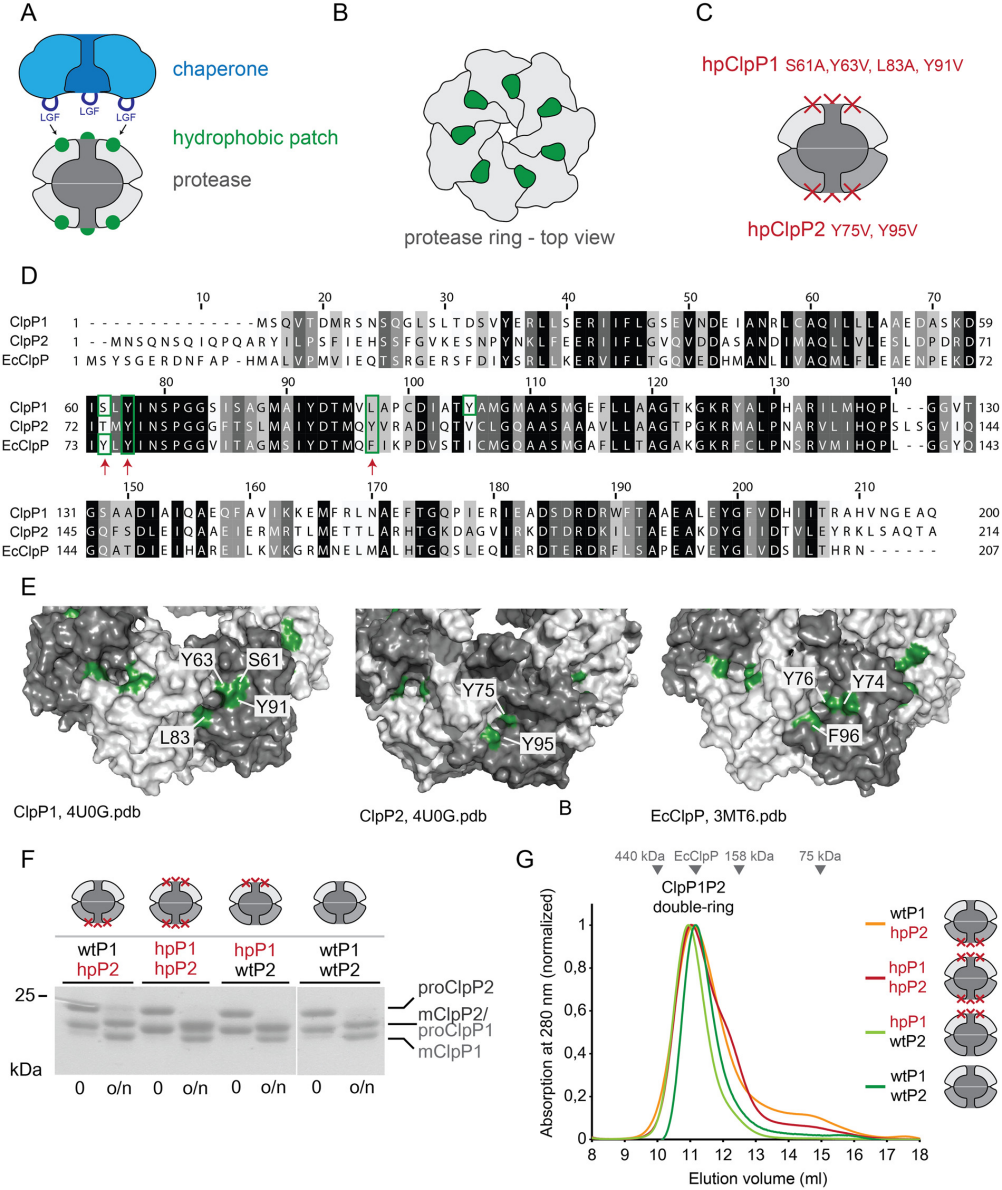
Generation of hydrophobic patch variants of ClpP1 and ClpP2. A. Cartoon representation of the LGF-loops (dark blue) of the chaperone binding to hydrophobic surface patches (green) on the protease core (grey). B. Top view of a heptameric protease ring. The hydrophobic patches (green) are formed by residues of two adjacent protease subunits (grey). C. Mutations introduced in ClpP1 and ClpP2 to create the hydrophobic patch variants hpClpP1 (ClpP1^S61A, Y63V, L83A, Y91V^) and hpClpP2 (ClpP2^Y75V, Y95V^). D. Alignment of Mtb ClpP1 and ClpP2 with EcClpP. Conservation is colored from white (not conserved) to black (identical). The identity between ClpP1/ClpP2 is 39.5%, between EcClpP/ClpP1 46% and EcClpP/ClpP2 44.4%. Red arrows highlight the residue positions of the EcClpP hydrophobic patch residues. Residues depicted in green in panel E are marked with a green box. E. Surface representation of ClpP1, ClpP2 (4U0G.pdb) and EcClpP (3MT6.pdb) ring faces. Individual subunits are colored alternatingly in light and dark grey. Hydrophobic patch residues are colored in green and labelled accordingly. For ClpP1 and ClpP2 the hydrophobic patch residues used for mutation are shown, for EcClpP reference residues are shown as described in the literature [14]. F. Creation of a set of mature mixed wild-type ClpP1 and ClpP2 (wtP1, wtP2) and hydrophobic patch variants (hpP1, hpP2). Large-scale processing of ClpP1 and ClpP2 (70 μM protomer each) containing the N-terminal propeptide (proClpP1, proClpP2) to mature ClpP1 and ClpP2 (mClpP1, mClpP2) in the presence of 1 mM activator. The reaction was performed in Buffer A. All samples were run on the same gel. The lane containing the size marker was removed for better visual representation (white line). G. Analytical gel filtration was performed with the mature ClpP1P2 complexes created in panel F. The peak at 11 ml shows that all complexes have assembled into double-rings.

To answer this question we aimed to create variants of ClpP1 and ClpP2 with impaired chaperone binding. One of the two important interaction features of the Clp proteases is a hydrophobic patch on the ClpP cylinder face, responsible for binding a loop on the chaperone containing a conserved LGF motif (Fig 3A). These hydrophobic patches are located in clefts on the apical surface of the ClpP particle and are formed by residues of two adjacent subunits (Fig 3B). To impair chaperone binding to the protease cylinder, we mutated the hydrophobic patch and termed the resultant proteins hydrophobic patch (hp) variants (hpClpP1and hpClpP2) in contrast to wild-type (wt) ClpP1 and ClpP2 (Fig 3C, impaired ClpP faces are denoted with red crosses). Conserved residues located in this patch have been described for EcClpP, namely Y74, Y76 and F96 [14]. To identify the corresponding residues in ClpP1 and ClpP2 we aligned the protein sequences of the three proteins (Fig 3D, red arrows) and also ascertained that the selected residues are located on the surface of the Mtb ClpP1P2 particle as judged from the published crystal structure (Fig 3E) [24]. To generate hpClpP1, four residues on the ClpP1 ring face were mutated: S61A, Y63V, L83A, Y91V. To generate hpClpP2, two residues were mutated: Y75V, Y95V (Fig 3C). Expression and purification of either variant resulted in a stable protein preparation.

From the wild-type subunits and the hpClpP variants, four different proClpP1P2 particles were produced, with either both partners wild-type (wtClpP1/wtClpP2), both partners hp variants (hpClpP1/hpClpP2), or one partner wild-type and the other hp variant in the two mixed particles (wtClpP1/hpClpP2 and hpClpP1/wtClpP2). For the generation of the mature particles, the proClpP1P2 particles were incubated in the presence of the activator and processing was verified by SDS-PAGE analysis. Fig 3F shows that processing to the mature particle occurs for all four particles. The correct size of the resulting mature particles was verified by mass spectrometry. Subsequent analytical gel filtration shows that all particles are assembled into the double-ring complex (Fig 3G). The peak fractions corresponding to the assembled particle were collected and the mature ClpP1P2 particles were used for subsequent experiments. Together, these results show that the hydrophobic patch mutations did not affect the assembly or peptidase function of the ClpP1P2 complexes, as particles containing hpClpP1 and hpClpP2 variants assemble to their double-ring functional state and are active in propeptide cleavage.

The interaction competence of the hydrophobic patch variants with the ATPase partners ClpX and ClpC1, both from Mtb, was then tested by chaperone-dependent protein degradation assays with ssrA-tagged model substrates. To assess ClpC1-dependent degradation, GFP carrying the Mtb ssrA-tag at its C-terminus (GFP-ssrA) was used, allowing detection of the activity by fluorescence spectroscopy. To measure ClpX-dependent degradation, malate dehydrogenase, extended C-terminally with the Mtb ssrA-tag (MDH-ssrA) was employed as a model substrate, because unfolding of the stable GFP-ssrA is not well supported by the weaker ClpX unfoldase activity.

**Fig 4:**
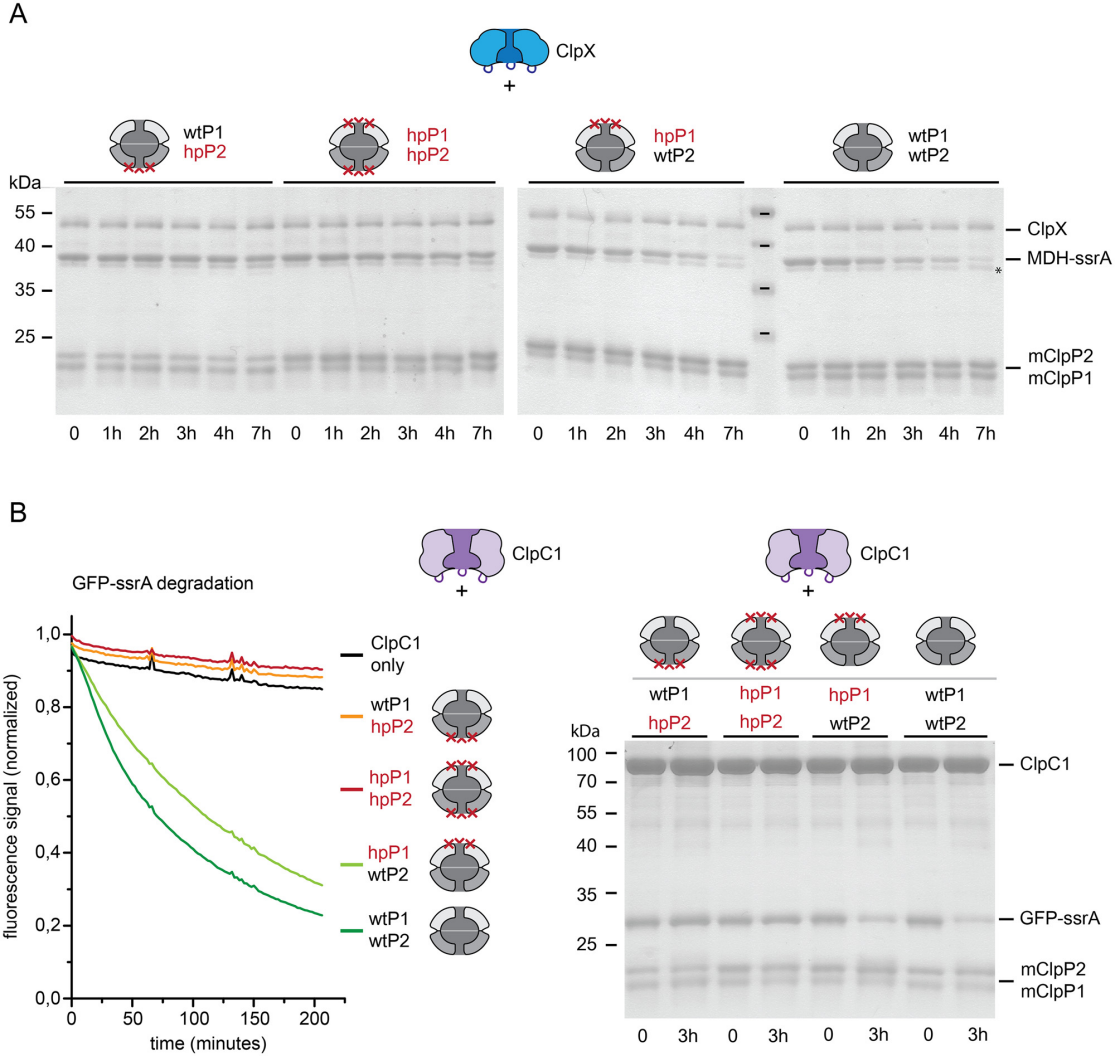
Chaperone-mediated degradation of ssrA-tagged substrates by ClpP1P2 requires the hydrophobic patch on ClpP2. ClpX and ClpC1-dependent degradation of model substrates was assayed with a set of mature ClpP1P2 particles, created from mixed wild-type (wtP1, wtP2) and hydrophobic patch variants (hpP1, hpP2) of ClpP1 and ClpP2. A. Degradation of MDH-ssrA (2 μM) mediated by ClpX (1 μM hexamer) and wt, hp or mixed mature ClpP1P2 particles (0.5 μM double-ring particle), was followed by the disappearance of the MDH-ssrA band in SDS-PAGE. The band just below MDH-ssrA that is not degraded (*) was confirmed by MS/MS to be composed of MDH, most probably lacking the ssrA tag. B. Degradation of GFP-ssrA (2 μM) mediated by ClpC1 (1 μM hexamer) and by wt, hp and mixed mature ClpP1P2 particles (0.5 μM double-ring particle) was monitored by the loss of the intrinsic GFP fluorescence signal. The signal was globally normalized. Additionally, time points were taken at the beginning and the end of the reaction and degradation of GFP-ssrA was confirmed by SDS-PAGE.

Fig 4A shows ClpX-dependent degradation of MDH-ssrA, measured by the disappearance of the MDH-ssrA protein band in SDS-PAGE. The wild-type complex supports almost complete degradation of MDH-ssrA over the time course of 7 hours. The small band below the MDH-ssrA band (marked with *) was confirmed by mass spectrometry to be MDH, most probably lacking the ssrA tag. As untagged MDH is not recruited to the ATPase, it can consequently not be degraded by the complex. As expected, the particle formed from both hydrophobic patch variants (hpP1/hpP2) does not exhibit any degradation of MDH-ssrA within the same time frame. Carrying out the same assay with the hydrophobic patch/wild-type mixed particles produces two opposite outcomes. With the particle in which ClpP2 carries the mutation (wtP1/hpP2), MDH-ssrA degradation is completely abolished, while the particle composed of a wild-type ClpP2 and a mutant ClpP1 ring (hpP1/wtP2) exhibits degradation activity comparable to the wild-type particle (Fig 4A). This demonstrates that ClpX-dependent degradation only occurs in context of the wild-type ClpP2 ring surface, indicating that ClpP2 is the interaction platform for association with ClpX.

ClpC1-dependent degradation of GFP-ssrA was then tested using the four different ClpP double-ring particles. Here, the degradation time course was measured by following the loss of the intrinsic fluorescence signal of GFP, and end point samples were assessed by SDS-PAGE analysis (Fig 4B). Analogous to ClpX-mediated degradation, we again observe degradation with the wild type particle (wtP1/wtP2) and the mixed particle carrying interaction-competent ClpP2 subunits (hpP1/wtP2). No degradation is observed for the hp particle (hpP1/hpP2) or the mixed particle carrying a mutation in the ClpP2 hydrophobic patch (wtP1/hpP2). Hence, ClpC1-dependent degradation also relies on ClpP2 as the interaction platform. Even in a heterologous setup, where EcClpX is used in place of Mtb ClpX, the particle with wild-type ClpP1 and a hydrophobic patch mutation in ClpP2 results in loss of activity, while the mixed particle carrying mutated ClpP1 and wild-type ClpP2 is fully active (S3 Fig). These results suggest that ClpP2 generally functions as the interaction platform for chaperone binding partners and that the Mtb ClpP1P2 particle in contrast to *E*. *coli* ClpP forms asymmetric complexes, capped only on one side by a chaperone partner.

All chaperone-dependent degradation assays were carried out in absence of the activator. Addition of the activator leads to a mild increase in the reaction rate, but does not change the overall result (S4 and S5 Figs). This means that also in the presence of activator, the wild-type ClpP2 interaction surface is required to obtain ClpX- or ClpC1-dependent substrate degradation.

Taken together, our results show that both chaperones of the Mtb Clp system, ClpX and ClpC1, need the interaction surface of ClpP2 to support degradation of ssrA-tagged substrates, indicating that ClpP2 is the interaction platform for both ClpX and ClpC1.

## Discussion

The Clp chaperone-protease complexes are formed by the coaxial stacking interaction of hexameric ATPase rings on top of the ClpP double-ring particle. In the well-studied *E*. *coli* Clp system, ATPase partners bind to both sides of the protease core to form symmetric particles [32, 33]. However, as we show here, in the Mtb Clp system this symmetric stacking does not occur. In contrast to *E*. *coli*, Mtb harbors not only one ClpP subunit, but two (ClpP1 and ClpP2), forming a hetero ClpP1P2 double-ring that presents two different chaperone interaction surfaces. While first theories proposed that each of the two chaperones, ClpX and ClpC1, specifically binds to either ClpP1 or ClpP2, our results clearly show that both chaperones only use the ring surface of ClpP2 to build the protein degradation-competent complexes.

There are two motifs on the ClpP protease core involved in chaperone interaction, the N-loop and a hydrophobic surface patch [14, 17–21]. In order to investigate interaction of both chaperones with the hetero double-ring protease particles, we designed mutants of ClpP1 and ClpP2 where the respective hydrophobic patches were mutated, thereby impairing chaperone binding. Indeed, ClpP1P2 particles where both proteases have the mutated hydrophobic patch are no longer able to support chaperone-mediated degradation. As this particle assembles into the double-ring complex and shows propeptide processing activity, we can be confident that the hydrophobic patch mutations neither impair correct ClpP1P2 complex formation nor do they affect the integrity of the active sites. Our selection of hydrophobic patch residues was based on three aromatic residues described for *E*. *coli* ClpP Y74, Y76 and F96 [14]. Mtb ClpP2 has two aromatic residues in corresponding positions, Y75 and Y95, that were selected for mutation, as they both were shown to coordinate ADEP, an antibiotic that binds into the hydrophobic patch and mimicks binding of the LGF-loop of the chaperone [24]. In ClpP1, only one of the three corresponding residues is an aromatic residue, Y63, so a nearby aromatic residue that stacked onto Y63, Y91, was also mutated, as well as two less bulky residues. As the hydrophobic patches of ClpP1 and ClpP2 are different in amino acid composition, they also differ slightly in shape. While ADEP binds into the hydrophobic patch of ClpP2, it is too bulky to fit into the hydrophobic patch of ClpP1 [24], suggesting that a similar reason might cause the LGF-loops of ClpX and ClpC1 (as well as the homologous IGF-loop of EcClpX) to only interact with ClpP2.

The second chaperone-binding element of the Clp proteases could also contribute to the differential interaction of ClpX and ClpC1 with ClpP2. The N-loops of ClpP1 and ClpP2 differ in their length, about 7 residues in ClpP1 versus 17 residues in ClpP2, and do not show structural similarities. The N-loop of ClpP2 is well resolved in the crystal structure, and contains a β-hairpin necessary for efficient substrate translocation [17, 24, 41], while the ClpP1 N-loop residues are resolved neither in the ClpP1P1 nor the ClpP1P2 structure [24, 42], indicating a large degree of flexibility. While the N-loops are not resolved in ClpP1 in either structure, the apparent axial pore size of ClpP1 in the inactive ClpP1P1 structure (12 Å) differs substantially from the one of ClpP1 in the active ADEP-bound ClpP1P2 structure (30 Å), indicating that ADEP-binding to ClpP2 allosterically opens the ClpP1 pore [24]. Therefore, binding of the ClpC1 and ClpX chaperones to ClpP2 might lead to an open ClpP1 pore—a situation that would be detrimental to the cell, as ADEP-activated ClpP was shown to degrade nascent protein chains [4]. However, it is unlikely that ClpP1 exists in the cell in a deregulated open-pore form. There are indications that pore widening by the chaperone binding partner is less pronounced than pore opening caused by ADEP-binding [43], in which case the ClpP1 pore in a chaperone-bound ClpP1P2 complex might not be as wide as observed in the ADEP-bound crystal structure. Furthermore, the active conformation of the ClpP1P2 complex in absence of activator is stimulated only in the presence of chaperone together with protein substrate [31]. Therefore it is possible that *in vivo* the ClpP1 pore is only open while substrate translocation from the ClpP2 side of the complex takes place, such that the chamber is filled with the translocating substrate chains and peptide products, preventing access of proteins from the ClpP1 side. An open or dynamic ClpP1 pore could thus rather present a pathway for product release, where exiting peptides might even prevent entrance of substrates. Alternatively, the flexible, in the structure unresolved, N-loops of ClpP1 could act as a kind of pore plug and adopt a “down” conformation, restricting access to the chamber from the ClpP1 side [18, 44].

The N-loop region is well conserved throughout ClpP2 homologues from various Actinobacteria (S7 Fig), and also shares residue identity with ClpP from *E*. *coli*, for example a conserved proline (S7 Fig, yellow box), which was shown to be important for maturation of ClpP and for ClpAP complex formation in *E*. *coli* [18]. This proline is not present in ClpP1 and most of its homologues from Actinobacteria (S6 Fig), and generally, the region that could form an N-loop is less conserved in ClpP1. In addition to the N-loop, the hydrophobic patch residues are also well conserved in ClpP2 (S7 Fig), and less well in ClpP1 (S6 Fig). The different pattern of conservation in ClpP1 and ClpP2 of the chaperone interaction elements suggests that the specialization of ClpP1 and ClpP2 occurs not only in Mtb, but is a general property of actinobacterial ClpP proteases. The asymmetry in interaction with ATPase partners of the ClpP particles might even extend to other gram-positive organisms, as EM reconstructions for the *Listeria monocytogenes* ClpP1P2 complex show a protruding N-terminal density only on ClpP2, which is reminiscent of the well resolved N-loop of ClpP2 in the Mtb ClpP1P2 structure [24, 45].

The conservation of the chaperone interaction motifs in ClpP2 along with our experimental results support the notion that ClpP2 is the canonical subunit involved in chaperone binding, while ClpP1 developed a more varied interaction surface throughout different organisms. If in the assembled chaperone-protease complex both ClpX and ClpC1 bind to ClpP2, this raises the question why both chaperones asymmetrically interact with the ClpP1P2 complex and only bind to one side of the cylinder, when it was shown for the *E*. *coli* Clp system that symmetric interaction increases the efficiency of the complex [32]. A possible competition between ClpX and ClpC1 for protease binding sites most likely poses no problem, since the protease subunits were shown to be amongst the most abundant proteins in the Mtb cell [46]. Binding of both chaperone partners to ClpP2 would leave the interaction surface of ClpP1 free for as of yet unknown putative interaction partners. A fragment of ADEP was for example shown to activate ClpP1, indicating that in principle binding is still possible [24].

Apart from the asymmetry in chaperone interaction, the Mtb ClpP1P2 complex also shows specialization in its processing activity of the ClpP1 and ClpP2 propeptides, as suggested by coexpression experiments of Mtb ClpP1 and ClpP2 in *E*. *coli* [29]. We show that the propeptide of ClpP2 is processed first, and that the processing activity is performed mostly by ClpP1. This differential processing could occur due to the length and/or the sequence of the propeptides. With 12 residues the ClpP2 propeptide has almost double the size of the ClpP1 propeptide (~7 residues) [29, 30], which, depending on the conformation of the putative ClpP1 loop residues, may not reach the active sites of ClpP2. Furthermore, the ClpP1 and ClpP2 active sites have different cleavage specificities and ClpP1 was shown to be especially important for cleavage after hydrohphobic residues, while ClpP2 was not well able to cleave after such a model peptide, which could contribute to a different role in propeptide cleavage [30]. We also show that the synthetic activator peptide is not necessary for propeptide processing, but that the presence of the natural interaction partners, chaperone and substrate, is sufficient for this reaction, suggesting that this is how processing is performed *in vivo*. The ATP-dependence of this reaction shows that chaperone assembly and chaperone-dependent unfolding and translocation of substrates is necessary for ClpP1P2 activation. Substrate binding in or near the active site could stabilize the active conformation of ClpP1P2 in a similar manner as the activator, and act synergistically with chaperone binding to stimulate ClpP1P2 activity [31]. *In vitro*, propeptide processing still seemed more complete in presence of the activator, presumably because the activator provided better long-term stabilization of the active ClpP1P2 conformation as it is not degraded.

During propeptide cleavage the assembled ClpP1P2 complex is stable at room temperature, as an excess of either subunit did not lead to more processing. This shows that the ClpP1P2 complex does not dissociate to reassociate with unprocessed excess subunits, and also that the processing is an intra-particle reaction. The ClpP1P2 complex in presence or absence of the propeptides forms readily at room temperature, without the activator peptide (as opposed to [30]), but dissociates at 4°C, indicating that the interaction between the ClpP1 and ClpP2 rings is mainly mediated by hydrophobic interactions [47–49]. Correlating hydrophobic interactions between the rings to the available structural information is not straightforward. The ClpP double-ring can adopt active/extended or inactive/compressed conformations with large differences in the interaction interface as represented by the active ClpP1P2 versus the inactive ClpP1P1 structure (S8 Fig) [24, 42]. Our assembly tests were performed in the absence of activator, and therefore the ClpP1P2 complex is presumably in an inactive conformation, as for activity either activator or chaperone together with substrate were shown to be necessary [30, 31]. Analysis of the interface residues between the ClpP1P2 rings and the ClpP1P1 rings shows that irrespective of the difference in the interaction surface area, both interfaces are composed in large portions of hydrophobic interactions that could account for the observed behavior (S9 Fig). However, neither of the complexes exactly represents the interface in our particle and a structure of the inactive conformation of ClpP1P2 would be required for a more quantitative analysis.

Under *in viv*o conditions the ClpP1P2 complex is most likely stable and in an inactive conformation, while the presence of chaperone and substrate dynamically activate the complex at need [31]. It remains to be seen, whether additional factors can regulate the activity and substrate specificity of the fully assembled Clp chaperone-proteases, potentially not only by binding to the ATPase rings as has been observed for various adaptors in other Clp proteases systems, but by binding to the available ClpP1 ring surface in the complex.

## Supporting Information

S1 Fig Negative-stain TEM micrograph of proClpP1. 1.4 μM proClpP1 (protomer) was stained with 2% aqueous uranyl acetate.
(TIF)

S2 Fig mClpP1 has a mass weight corresponding to processing after Met7. Electron spray ionisation mass spectrometry of mClpP1P2. proClpP1P2 was processed overnight in the presence of 1 mM activator to produce the mature complex. The expected mass for mClpP1-His_4_ processed after Met7 is 21463.3 Da. For mClpP2-His_4_ processed after Ala12 the expected mass is 22760.9 Da.
(TIF)

S3 Fig EcClpX-mediated degradation of GFP-ssrA by ClpP1P2 requires the hydrophobic patch on ClpP2. EcClpX-mediated (1 μM hexamer) degradation of GFP-ssrA (*E*. *coli* ssrA tag sequence) (2 μM) by wild-type (wt), hydrophobic patch (hp) and mixed mature ClpP1P2 particles (0.5 μM double-ring particle) was monitored by the loss of the intrinsic GFP fluorescence signal. The signal was globally normalized.
(TIF)

S4 Fig ClpC1-mediated degradation of GFP-ssrA by ClpP1P2 requires the hydrophobic patch on ClpP2 also in presence of activator. ClpC1-mediated degradation (1 μM hexamer) of GFP-ssrA (2 μM) by wild-type (wt), hydrophobic patch (hp) and mixed mature ClpP1P2 particles (0.5 μM double-ring particle) in the presence of 1 mM activator was monitored by the loss of the intrinsic GFP fluorescence signal. The signal was globally normalized.
(TIF)

S5 Fig ClpX-mediated degradation of MDH-ssrA by ClpP1P2 requires the hydrophobic patch on ClpP2 also in presence of activator. ClpX-mediated degradation (1 μM hexamer) of the substrate MDH-ssrA (2 μM) by wild-type (wt), hydrophobic patch (hp) and mixed mature ClpP1P2 particles (0.5 μM double-ring particle) in the presence of 1 mM activator, was followed by the disappearance of the MDH-ssrA band on an SDS-PA gel at the time points indicated below the gel.
(TIF)

S6 Fig Sequence conservation amongst Mtb ClpP1 and actinobacterial homologues. ClpP1 was aligned with homologous actinobacterial proteins. Conservation is colored from white (not conserved) to black (identical). Sequences and naming were extracted from the Uniprot database. The designation of ClpP subunits as ClpP1 or ClpP2 for different Actinobacteria does not always match the Mtb designation. Alignment was based on homology, not on the naming of the subunits. The Uniprot identifiers are given in the sequence labels. Organism abbreviations: ARTAT: *Arthrobacter aurescens*, BIFLO: *Bifidobacterium longum*, CORGL: *Corynebacterium glutamicum*, MICLC: *Micrococcus luteus*, MYCS2: *Mycobacterium smegmatis*, MYCTU: *Mycobacterium tuberculosis*, NOCFA: *Nocardia farcinica*, PROAC: *Propionibacterium acnes*, RHOJR: *Rhodococcus jostii*, STRCO: *Streptomyces coelicolor*. The label for Mtb ClpP1 is colored in red, hydrophobic patch residues are marked with green boxes. The annotation of the N-loop and pore residues is based on the Mtb ClpP1 structure (2CE3.pdb).
(TIF)

S7 Fig Sequence conservation amongst Mtb ClpP2 and actinobacterial homologues. ClpP2 was aligned with homologous actinobacterial proteins. Conservation is colored from white (not conserved) to black (identical). Sequences and naming were extracted from the Uniprot database. The designation of ClpP subunits as ClpP1 or ClpP2 for different Actinobacteria does not always match the Mtb designation. Alignment was based on homology, not on the naming of the subunits. The Uniprot identifiers are given in the sequence labels. Organism abbreviations: ARTAT: *Arthrobacter aurescens*, BIFLO: *Bifidobacterium longum*, CORGL: *Corynebacterium glutamicum*, MICLC: *Micrococcus luteus*, MYCS2: *Mycobacterium smegmatis*, MYCTU: *Mycobacterium tuberculosis*, NOCFA: *Nocardia farcinica*, PROAC: *Propionibacterium acnes*, RHOJR: *Rhodococcus jostii*, STRCO: *Streptomyces coelicolor*. The label for Mtb ClpP2 is colored in red, hydrophobic patch residues are marked with green boxes and a conserved proline with a yellow box. The annotation of the N-loop and pore residues is based on the Mtb ClpP2 structure (4U0G.pdb).
(TIF)

S8 Fig The Mtb ClpP1P1 and ClpP1P2 show differences in their ring-ring interaction surface areas. Interaction residues of ClpP1P1 (2CE3.pdb, left side) and ClpP1P2 (4U0G.pdb, right side) were determined and depicted using the COCOMAPS web application with standard settings [50]. The individual rings are colored light violet and light pink in cartoon representation, while the respective interaction residues are colored in dark violet and dark pink and are additionally shown in stick representation.
(TIF)

S9 Fig Hydrophobic interactions in the ClpP1P1 and ClpP1P2 ring-ring interfaces. The interaction surface areas of ClpP1 (upper left) and ClpP2 (upper right) of the active/extended ClpP1P2 structure (4U0G.pdb) and of one ClpP1 ring (lower left) of the inactive/compressed ClpP1P1 structure (2CE3.pdb). The rings are shown from the interface side and the residues involved in the interaction, as determined by the COCOMAPS web application with standard settings [50] are rimmed with a yellow dotted line. Amino acids are colored according to their hydrophobicity using the Eisenberg hydrophobicity scale (http://web.expasy.org/protscale/pscale/Hphob.Eisenberg.html). Red color indicates the most hydrophobic and white color the least hydrophobic residues.
(TIF)
