# Correction: The *Mycobacterium tuberculosis* ClpP1P2 Protease Interacts Asymmetrically with Its ATPase Partners ClpX and ClpC1

**DOI:** 10.1371/journal.pone.0131132

**Published:** 2015-06-19

**Authors:** The PLOS ONE Staff

## Notice of Republication

This article was republished on May 22, 2015, to correct Fig. 1, which was distorted, and to remove hyphens that were incorrectly included in the words “chaperone” and “protein” in the first sentence of the Abstract. The publisher apologizes for the errors. Please download this article again to view the correct version. The originally published, uncorrected article and the republished, corrected article are provided here for reference.

## Supporting Information

S1 File Originally published, uncorrected article. (PDF)

S2 File Republished, corrected article. (PDF)
