# Supplementary figures and images for: The Mycobacterium tuberculosis ClpP1P2 Protease Interacts Asymmetrically with Its ATPase Partners ClpX and ClpC1
Source: PLoS One. 2015 May 1;10(5):e0125345. doi: 10.1371/journal.pone.0125345 (PMC4416901; doi:10.1371/journal.pone.0125345)

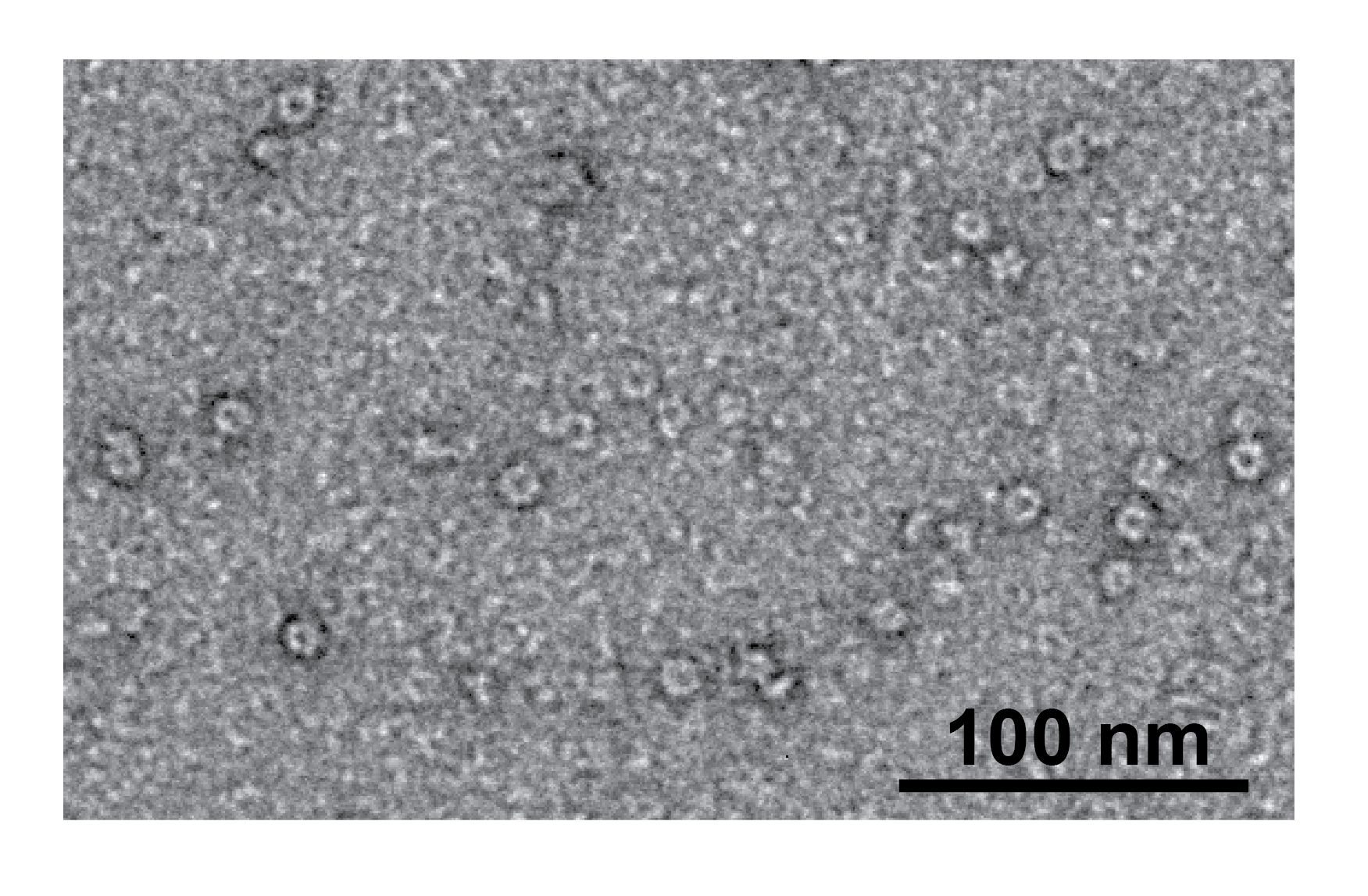

Supplement: S1 Fig — 1.4 μM proClpP1 (protomer) was stained with 2% aqueous uranyl acetate. (TIF) [file pone.0125345.s001.tif]

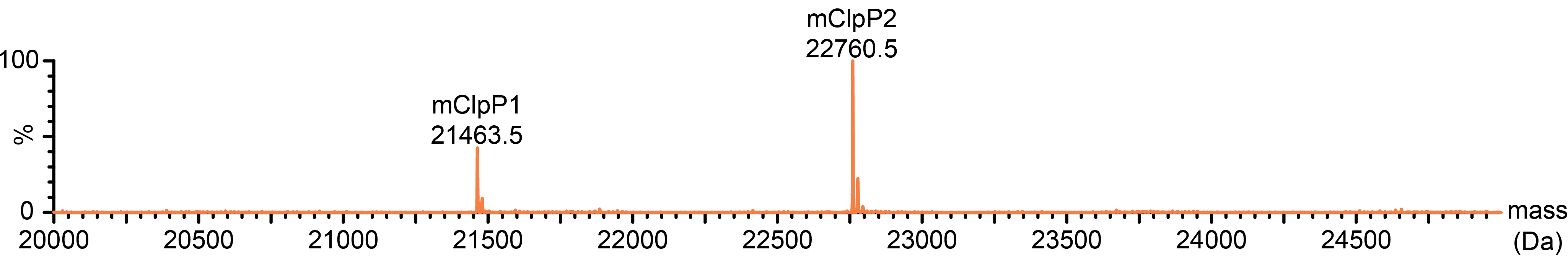

Supplement: S2 Fig — Electron spray ionisation mass spectrometry of mClpP1P2. proClpP1P2 was processed overnight in the presence of 1 mM activator to produce the mature complex. The expected mass for mClpP1-His4 processed after Met7 is 21463.3 Da. For mClpP2-His4 processed after Ala12 the expected mass is 22760.9 Da. (TIF) [file pone.0125345.s002.tif]

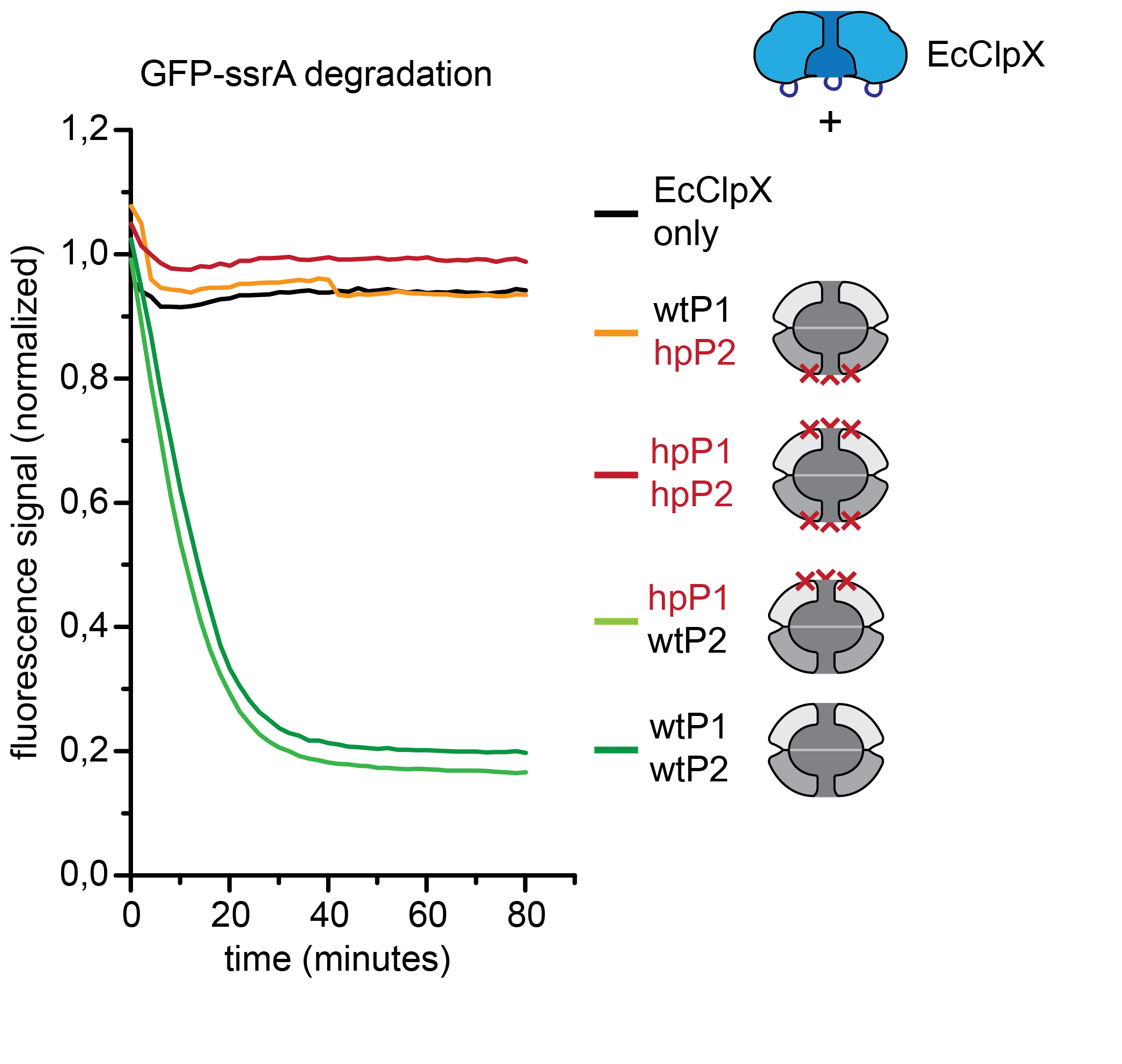

Supplement: S3 Fig — EcClpX-mediated (1 μM hexamer) degradation of GFP-ssrA (E. coli ssrA tag sequence) (2 μM) by wild-type (wt), hydrophobic patch (hp) and mixed mature ClpP1P2 particles (0.5 μM double-ring particle) was monitored by the loss of the intrinsic GFP fluorescence signal. The signal was globally normalized. (TIF) [file pone.0125345.s003.tif]

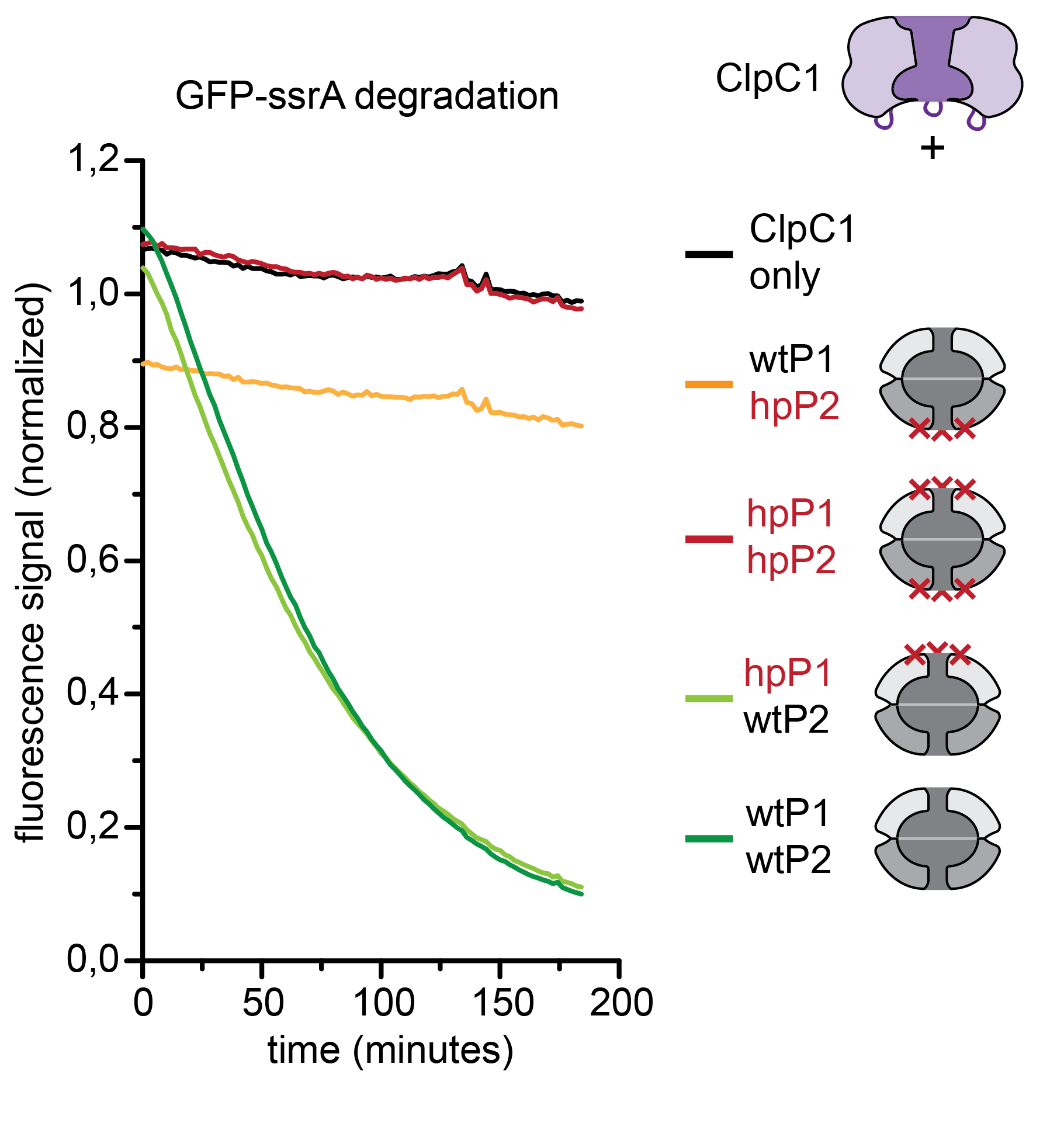

Supplement: S4 Fig — ClpC1-mediated degradation (1 μM hexamer) of GFP-ssrA (2 μM) by wild-type (wt), hydrophobic patch (hp) and mixed mature ClpP1P2 particles (0.5 μM double-ring particle) in the presence of 1 mM activator was monitored by the loss of the intrinsic GFP fluorescence signal. The signal was globally normalized. (TIF) [file pone.0125345.s004.tif]

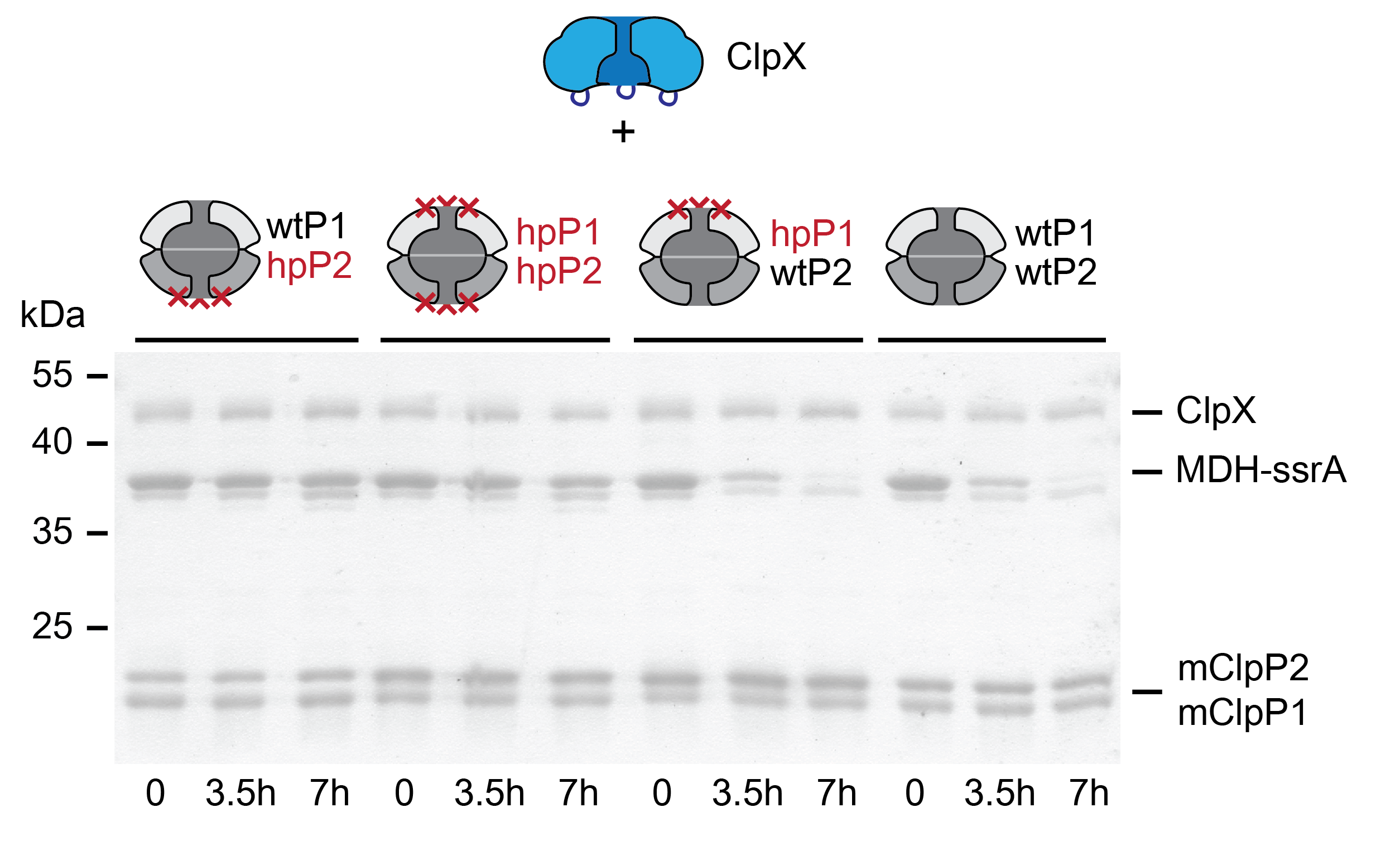

Supplement: S5 Fig — ClpX-mediated degradation (1 μM hexamer) of the substrate MDH-ssrA (2 μM) by wild-type (wt), hydrophobic patch (hp) and mixed mature ClpP1P2 particles (0.5 μM double-ring particle) in the presence of 1 mM activator, was followed by the disappearance of the MDH-ssrA band on an SDS-PA gel at the time points indicated below the gel. (TIF) [file pone.0125345.s005.tif]

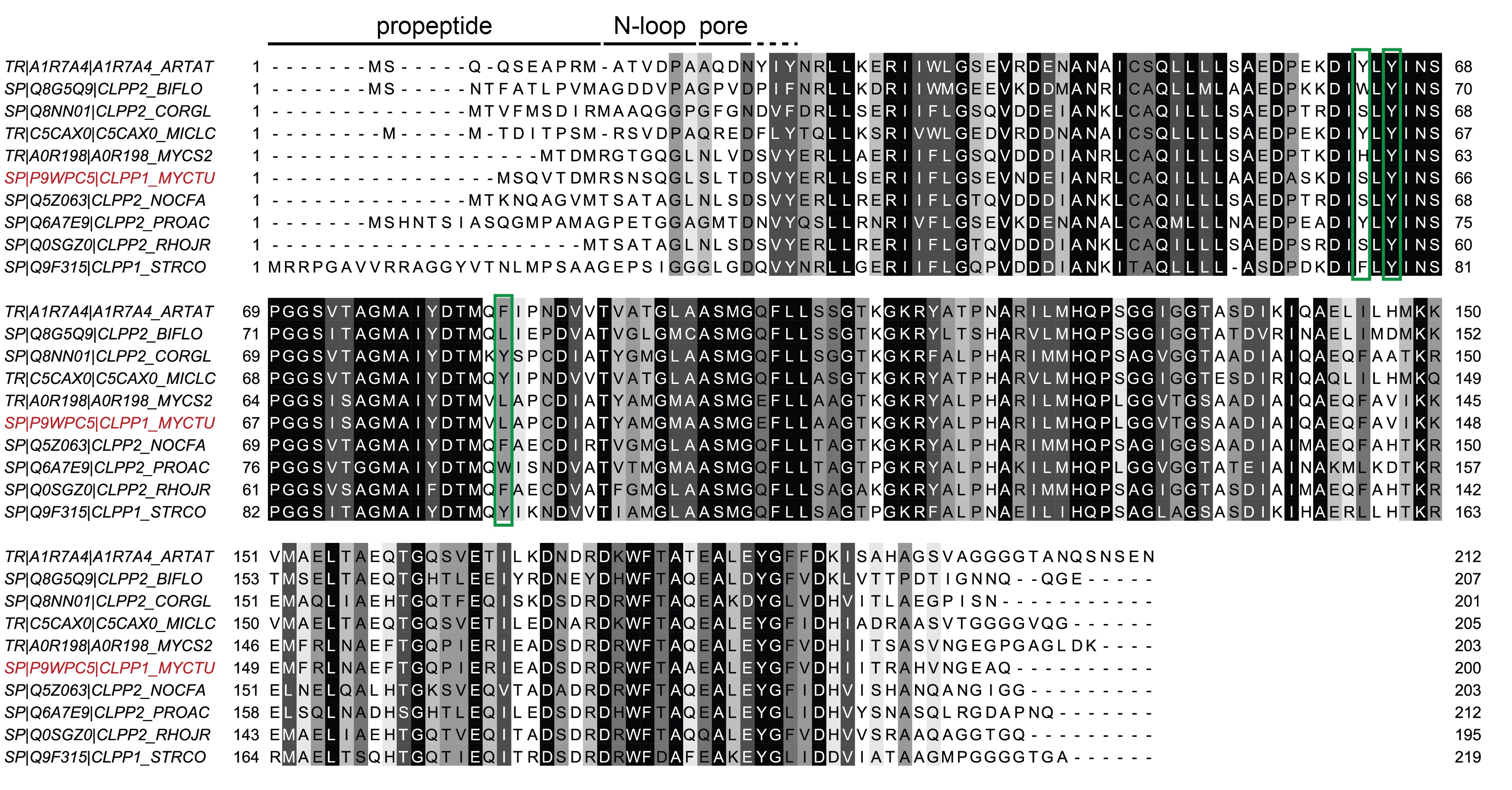

Supplement: S6 Fig — ClpP1 was aligned with homologous actinobacterial proteins. Conservation is colored from white (not conserved) to black (identical). Sequences and naming were extracted from the Uniprot database. The designation of ClpP subunits as ClpP1 or ClpP2 for different Actinobacteria does not always match the Mtb designation. Alignment was based on homology, not on the naming of the subunits. The Uniprot identifiers are given in the sequence labels. Organism abbreviations: ARTAT: Arthrobacter aurescens, BIFLO: Bifidobacterium longum, CORGL: Corynebacterium glutamicum, MICLC: Micrococcus luteus, MYCS2: Mycobacterium smegmatis, MYCTU: Mycobacterium tuberculosis, NOCFA: Nocardia farcinica, PROAC: Propionibacterium acnes, RHOJR: Rhodococcus jostii, STRCO: Streptomyces coelicolor. The label for Mtb ClpP1 is colored in red, hydrophobic patch residues are marked with green boxes. The annotation of the N-loop and pore residues is based on the Mtb ClpP1 structure (2CE3.pdb). (TIF) [file pone.0125345.s006.tif]

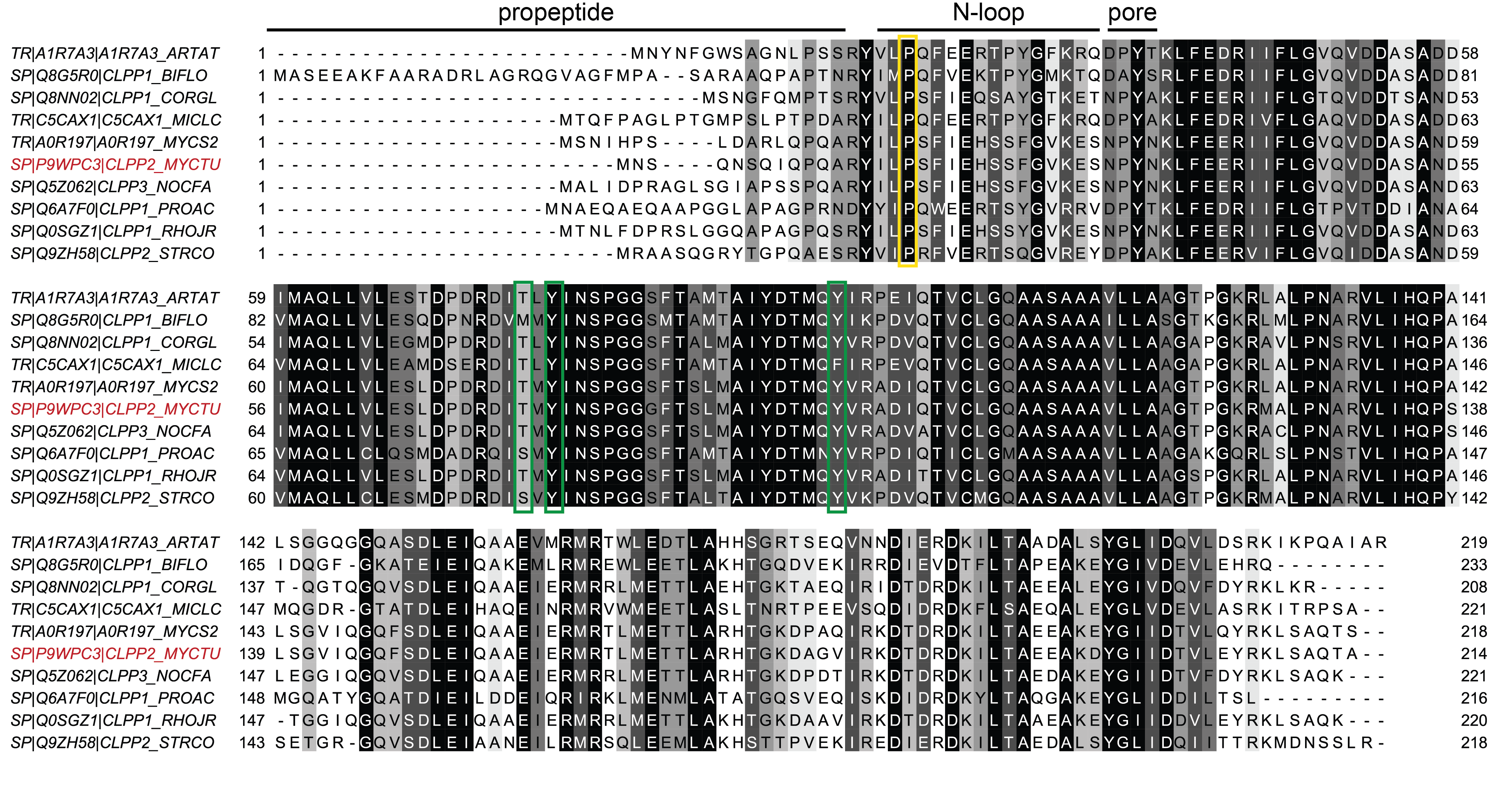

Supplement: S7 Fig — ClpP2 was aligned with homologous actinobacterial proteins. Conservation is colored from white (not conserved) to black (identical). Sequences and naming were extracted from the Uniprot database. The designation of ClpP subunits as ClpP1 or ClpP2 for different Actinobacteria does not always match the Mtb designation. Alignment was based on homology, not on the naming of the subunits. The Uniprot identifiers are given in the sequence labels. Organism abbreviations: ARTAT: Arthrobacter aurescens, BIFLO: Bifidobacterium longum, CORGL: Corynebacterium glutamicum, MICLC: Micrococcus luteus, MYCS2: Mycobacterium smegmatis, MYCTU: Mycobacterium tuberculosis, NOCFA: Nocardia farcinica, PROAC: Propionibacterium acnes, RHOJR: Rhodococcus jostii, STRCO: Streptomyces coelicolor. The label for Mtb ClpP2 is colored in red, hydrophobic patch residues are marked with green boxes and a conserved proline with a yellow box. The annotation of the N-loop and pore residues is based on the Mtb ClpP2 structure (4U0G.pdb). (TIF) [file pone.0125345.s007.tif]

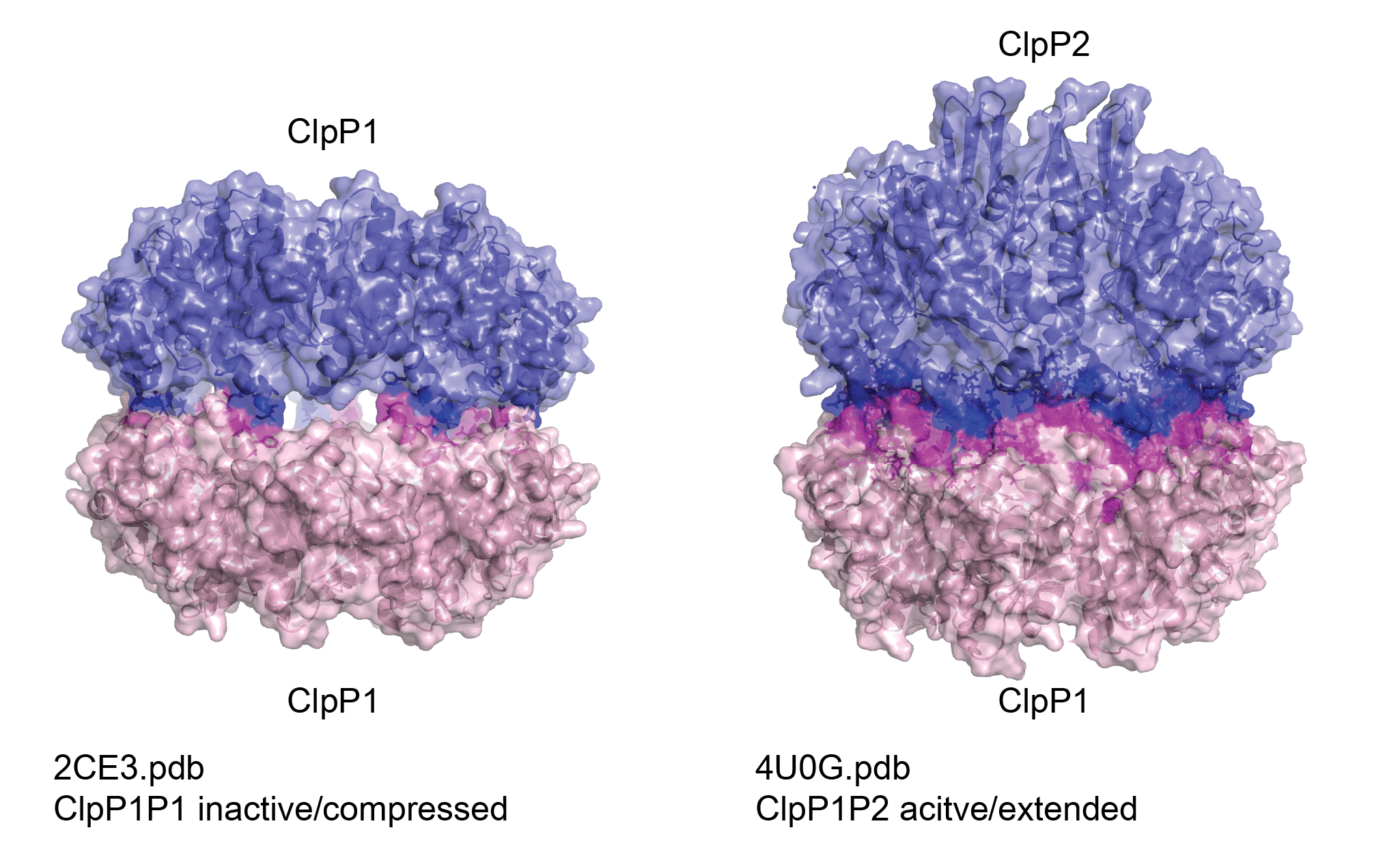

Supplement: S8 Fig — Interaction residues of ClpP1P1 (2CE3.pdb, left side) and ClpP1P2 (4U0G.pdb, right side) were determined and depicted using the COCOMAPS web application with standard settings [50]. The individual rings are colored light violet and light pink in cartoon representation, while the respective interaction residues are colored in dark violet and dark pink and are additionally shown in stick representation. (TIF) [file pone.0125345.s008.tif]

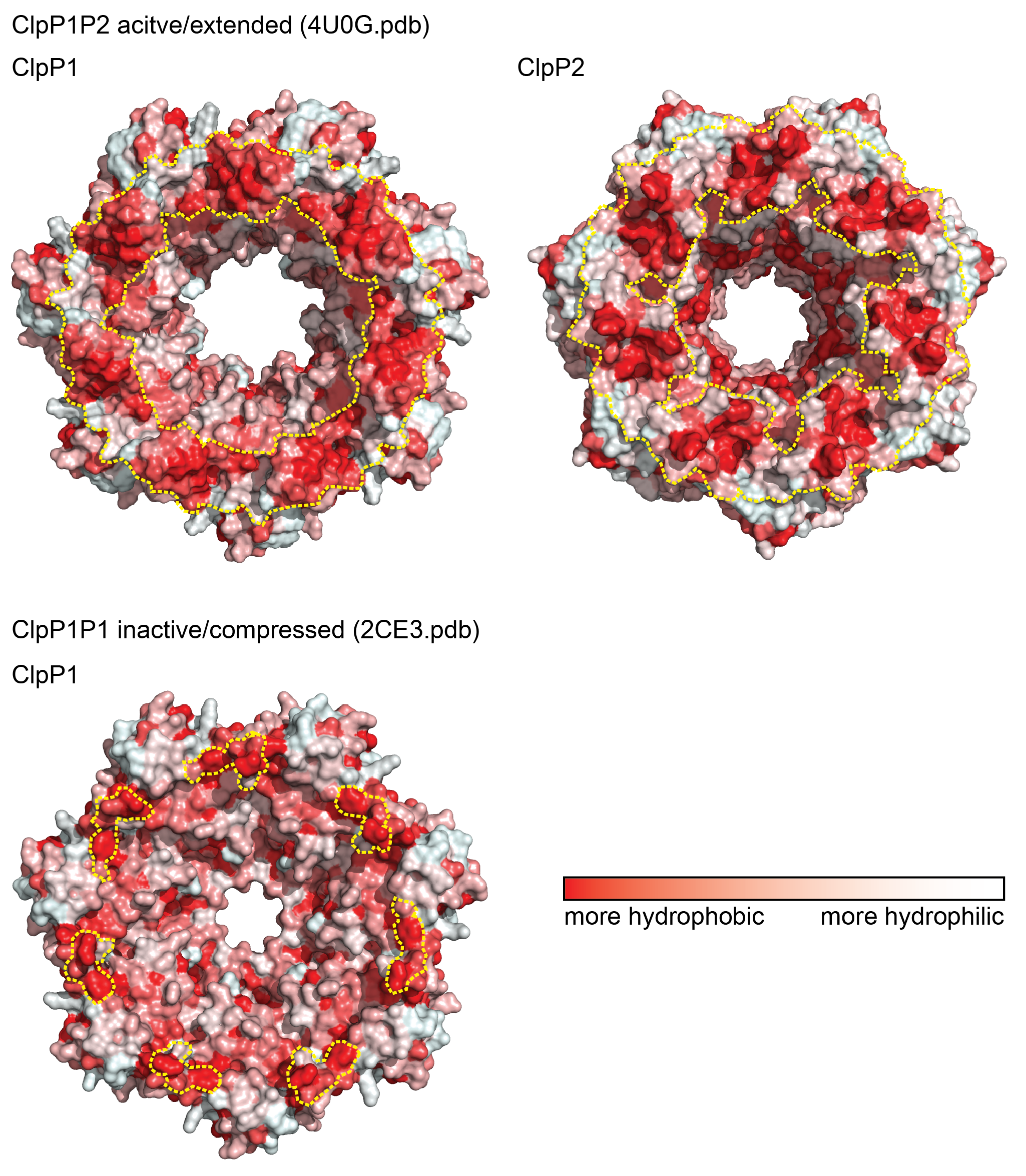

Supplement: S9 Fig — The interaction surface areas of ClpP1 (upper left) and ClpP2 (upper right) of the active/extended ClpP1P2 structure (4U0G.pdb) and of one ClpP1 ring (lower left) of the inactive/compressed ClpP1P1 structure (2CE3.pdb). The rings are shown from the interface side and the residues involved in the interaction, as determined by the COCOMAPS web application with standard settings [50] are rimmed with a yellow dotted line. Amino acids are colored according to their hydrophobicity using the Eisenberg hydrophobicity scale (http://web.expasy.org/protscale/pscale/Hphob.Eisenberg.html). Red color indicates the most hydrophobic and white color the least hydrophobic residues. (TIF) [file pone.0125345.s009.tif]
